# Exploring factors influencing the acceptance of ChatGPT in higher education: A smart education perspective

**DOI:** 10.1016/j.heliyon.2024.e31887

**Published:** 2024-05-25

**Authors:** Abeer S. Almogren, Waleed Mugahed Al-Rahmi, Nisar Ahmed Dahri

**Affiliations:** a Department of visual arts, College of arts, King Saud University, 11362, P.O.Box. 145111, Riyadh, Saudi Arabia; b Faculty of Social Science and Humanities, School of Education, Universiti Teknologi Malaysia, Skudai, 81310, Johor, Malaysia; c School of Computer Science and Engineering, Southeast University, Nanjing, China

**Keywords:** Smart education, ChatGPT, Higher education, Acceptance factors, Technology adoption

## Abstract

AI-powered chatbots hold great promise for enhancing learning experiences and outcomes in today's rapidly evolving education system. However, despite the increasing demand for such technologies, there remains a significant research gap regarding the factors influencing users' acceptance and adoption of AI-powered chatbots in educational contexts. This study aims to address this gap by investigating the factors that shape users' attitudes, intentions, and behaviors towards adopting ChatGPT for smart education systems. This research employed a quantitative research approach, data were collected from 458 of participants through a structured questionnaire designed to measure various constructs related to technology acceptance, including perceived ease of use, perceived usefulness, feedback quality, assessment quality, subject norms, attitude towards use, and behavioral intention to use ChatGPT. Structural model analysis (SEM) Statistical techniques were then utilized to examine the relationships between these constructs. The findings of the study revealed that Perceived ease of use and perceived usefulness emerged as significant predictors of users' attitudes towards ChatGPT for smart education. Additionally, feedback quality, assessment quality, and subject norms were found to positively influence users' behavioral intentions to use ChatGPT for smart educational purposes. Moreover, users' attitudes towards use and behavioral intentions were significantly proved for the actual adoption of ChatGPT. However, a few hypotheses, such as the relationship between trust in ChatGPT and perceived usefulness, were not supported by the data. This study contributes to the existing body information systems applications for the determining factor of technology acceptance in smart education context.

## 1 Introduction

The rapid transformation of education towards a technology-driven, personalized approach is undeniable. Smart education systems, characterized by their integration of cutting-edge tools and platforms, are revolutionizing how students learn and engage with course material [1]. These systems aim to cater to diverse learning styles, optimize learning outcomes, and foster a more immersive and accessible educational experience [2]. Smart education programs seek to use cutting-edge technology in the current digital world to improve student engagement, create conducive and inclusive learning environments that are easily accessible, and improve educational outcomes [1].

Recent research highlights the revolutionary potential of smart education initiatives in higher education. For instance, studies explore the transformative power of immersive technologies like Augmented Reality (AR) and Virtual Reality (VR) in boosting student engagement and knowledge retention [3]. These technologies enhance the educational experience by providing students with innovative ways to visualize complex concepts and engage in experiential learning. Similarly, Lin et al. [4] delve into the impact of Artificial Intelligence (AI) on smart education, emphasizing its role in intelligent tutoring and adaptive learning systems [4]. They posit that AI can personalize learning paths, offer real-time feedback, and identify areas for improvement, ultimately supporting self-directed learning and academic performance. The integration of Internet of Things (IoT) devices and sensor networks within educational settings is another promising avenue explored by Ref. [5], They emphasize the potential of these tools to gather real-time data on student interactions and learning behaviors, which can pave the way for personalized feedback and interventions, leading to improved learning outcomes and student satisfaction.

Generative Pre-trained Transformers (ChatGPT), a powerful AI-powered chatbot, has emerged as a promising tool within the smart education context. By simulating human-like conversations and supports the natural language processing, ChatGPT offers a unique opportunity to personalize learning experiences, provide students with on-demand support and feedback, and promote self-directed learning [6,7]. Previous studies have touched upon the diverse applications of ChatGPT in education. For instance, research by Rudolph et al. [8], highlights its potential to facilitate online discussions by enabling students to ask questions, clarify concepts, and receive course-related advice from the chatbot. Additionally [9], explored the use of ChatGPT for personalized tutoring, demonstrating its ability to provide students with customized support based on their individual needs and learning styles. A subfield of artificial intelligence called generative artificial intelligence uses deep learning to produce text, pictures, audio, and other media on its own [8,10,11]. According to academics, ChatGPT has a lot of uses in education, and its potential to transform teaching and learning is highly anticipated [12]. ChatGPT is thought to help teachers manage their tasks more efficiently and provide pupils more individualized instruction [13]. ChatGPT serves as an intelligent tutoring assistant, relieving instructors of tedious work so they may concentrate on developing students' critical thinking and abilities [14].

Additionally, ChatGPT has been used for academic advice, language acquisition, and tutoring in a variety of educational settings. The effectiveness of ChatGPT bots in language learning is examined by Ref. [15], who show how they may give learners contextualized language practice and engage them in meaningful discussions. ChatGPT enables real-time interaction between students and educational information by using machine learning and natural language comprehension, such as content production, cooperative problem solving, information search, and tutoring [16].

ChatGPT, as a key part of smart education ecosystems, has the potential to transform teaching methods, enhance educational opportunities, and promote student independence and self-directed learning. Fidan & Gencel [17], recent research looks on the use of ChatGPT bots in online course discussions. The researchers discovered that adding ChatGPT bots to discussion boards increased student engagement and promoted deeper exchanges amongst participants. In the online classroom, students felt more at ease asking questions and having conversations with the Chatbots, which improved their learning opportunities and fostered a sense of community. In a similar vein, few studies investigated ChatGPT's efficacy in offering students enrolled in beginning computer science courses individualized tutoring help. According to their research, students who engaged with the chatbots improved their course performance more than those who did not [12,18]. Higher levels of student satisfaction and engagement followed from the Chatbots' ability to customize their replies to each student's unique learning requirements and provide focused help.

Additionally, latest study from 2023 investigated ChatGPT's use in higher education institutions for academic advising reasons [19,20]. According to the survey, students who used the chatbots to get academic help expressed greater levels of support and satisfaction with the advising process. Students were able to make well-informed choices regarding their academic journeys because of the Chatbots' prompt advice on course selection, academic preparation, and career paths. Notwithstanding ChatGPT's potential advantages for higher education, a number of issues and concerns need to be considered to make sure it is used successfully [21]. For instance, given how sensitive student information is while interacting with chatbots, worries around data security and privacy can surface [6,7]. In order to keep consumers' confidence and legitimacy, it is also crucial to guarantee the precision and dependability of Chatbot answers. Furthermore, in order to improve usability and user experience, user-centered techniques should be given priority in the design and development of ChatGPT systems [22].

Additionally, latest study from 2023 investigated ChatGPT's use in higher education institutions for academic advising reasons [19,20]. According to the survey, students who used the chatbots to get academic help expressed greater levels of support and satisfaction with the advising process. Students were able to make well-informed choices regarding their academic journeys because of the Chatbots' prompt advice on course selection, academic preparation, and career paths. Notwithstanding ChatGPT's potential advantages for higher education, a number of issues and concerns need to be considered to make sure it is used successfully [21].

While the potential benefits of ChatGPT for smart education are vast, understanding the factors influencing its adoption is critical for successful implementation. Existing research on technology acceptance models (TAMs) has provided valuable understandings into user attitudes and behaviors towards various technologies [23]. However, limited research has specifically explored the factors influencing the acceptance of AI-powered chatbots like ChatGPT in educational contexts [24]. This gap in knowledge hinders educators and policymakers from maximizing the potential of ChatGPT for student learning. For instance, AI chatbots like ChatGPT introduce a novel layer of human-computer interaction within the learning environment. Users may have varying levels of experience and comfort with chatbot technology, potentially impacting their perceived ease of use and trust in ChatGPT for academic tasks.

Several recent studies have discussed the importance of ChatGPT and its adoption role in the education field. Dahri et al. [25], investigated student acceptance of ChatGPT for self-regulated learning, highlighting the importance of user-friendliness and functionalities that support independent learning strategies. Sánchez-Reina et al. [26] explored potential resistance towards AI in education, emphasizing the need to understand student concerns and promote positive attitudes. Abdaljaleel et al. [27] identified a positive correlation between perceived usefulness and student adoption, underlining the importance of demonstrating the practical benefits of ChatGPT in achieving learning objectives.

Furthermore, Choudhury & Shamszare [28], found that user trust in ChatGPT's accuracy and reliability is crucial for adoption. These studies, along with Bilquise et al. [29], Menon & Shilpa [23], Rathore [30], and Foroughi et al. [31], collectively showcase various applications of ChatGPT in education, including personalized learning, content creation, formative assessment, and 24/7 learning support.

While extensive research has explored user acceptance of various educational technologies, a significant gap exists regarding AI-powered chatbots like ChatGPT. TAMs typically explore factors like perceived ease of use, perceived usefulness, and compatibility with existing workflows [3]. However, applying these models directly to AI chatbots in education may not capture the nuances of user perceptions and interactions within this specific context [32]. Current research on the adoption of AI tools in education often focuses on general categories like "AI-powered learning systems" or "intelligent tutoring platforms." This study aims to address this gap by specifically investigating user acceptance of ChatGPT, a well-defined AI chatbot ChatGPT with unique functionalities. By examining the specific factors influencing the adoption of ChatGPT, we can provide educators with more targeted insights for integrating the tool into their teaching practices.

This study demonstrates the need to extend TAM frameworks when examining AI adoption in education. This research aims to address this knowledge gap by specifically exploring the factors influencing user acceptance of ChatGPT in higher education. By incorporating insights from recent studies and tailoring the approach to the unique context of AI chatbots, we can develop a more comprehensive understanding of user perceptions and ultimately promote successful integration of ChatGPT into smart education systems. These variables may include doubts about the technology's efficacy and usability, opinions about its utility and simplicity of use, compatibility with current teaching methods, and the system's ability to provide constructive criticism [33,34]. Furthermore, attitudes and intentions about the adoption of ChatGPT may be significantly influenced by individual traits as well as environmental circumstances. Nevertheless, a thorough grasp of these elements and how they interact in the context of higher education settings is lacking in the research that is currently available [33,34].

The main purpose of this research is to investigate the factors influencing user acceptance of ChatGPT for educational purposes in higher education settings. By employing a comprehensive research model informed by Technology Acceptance Models (TAMs) and incorporating additional relevant constructs, this study aims to achieve the following specific objectives:

1. Examine the influence of individual user perceptions on the adoption of ChatGPT, including trialability, perceived compatibility, relative advantage, and trust in ChatGPT.
2. Evaluate the impact of interaction quality with ChatGPT on user perceptions, focusing on feedback quality and perceived assessment quality.
3. Explore the role of social influence in shaping user attitudes towards ChatGPT, investigating the impact of subject norms.
4. Develop a comprehensive understanding of the relationships between the proposed constructs and their influence on key user outcomes: perceived usefulness, perceived ease of use, attitude towards using ChatGPT for education, behavioral intention to use, and overall acceptance of ChatGPT for education.

By achieving these objectives, this research will contribute valuable insights to the growing body of knowledge surrounding AI adoption in education. The findings will inform strategies for promoting the successful integration of ChatGPT in higher education by addressing factors that influence user acceptance and ultimately optimize the learning experience for both students and educators.

## 2 Technology acceptance models for AI adoption

Researchers are examining the variables impacting consumers' acceptance and use behaviors of artificial intelligence (AI) technologies as a result of the widespread deployment of these technologies in a variety of fields, including smart education systems. In order to know the adoption of AI applications, a number of technological acceptance models have been put forward [34,35]. These models provide helpful frameworks for examining user attitudes, perceptions, and intentions towards these technologies.

The Unified Theory of Acceptance and Use of Technology (UTAUT), which combines components from many other models to describe technology adoption behavior, is one of the most often utilized models in this field. The UTAUT paradigm has been used in recent research to examine the uptake of AI in educational environments [36,37]. For instance, S. Chen et al. used the UTAUT model to study the variables impacting instructors' willingness to use AI-driven educational tools. Their research showed that instructors' views and intentions about the adoption of AI were highly impacted by performance expectation, effort expectancy, and enabling factors [38,39]. Dahri et al. [25], investigated student acceptance of AI-based academic support in Malaysian and Pakistani universities.

Their model explores factors influencing this adoption, including perceived effectiveness (performance expectancy), ease of use (effort expectancy), information accuracy, and alignment with learning styles (pedagogical fit). They found that students who believe the AI system can improve their performance and is easy to use are more likely to accept it. The study suggests that well-designed AI academic support systems can enhance student learning. Comparably, a lot of work has been done to investigate consumer adoption of AI technology using the Technology adoption Model (TAM) and its derivatives. An enhanced TAM framework was used in a recent study by Page and Gehlbach [40], to examine the use of AI-driven virtual assistants in higher education. Their research revealed that students' views and intentions about the employment of virtual assistants for academic assignments were strongly impacted by perceived usefulness, simplicity of use, and trust. The research also emphasized how important perceived fun and social impact are in influencing how students see AI technology [40].

According to recent studies, people's attitudes towards AI technology and their desire to interact with them are greatly influenced by their level of trust. For example, research conducted [13] looked at how customers' adoption of AI-powered virtual assistants in e-commerce environments is influenced by trust. The results showed that consumers' views and intentions towards adopting virtual assistants for shopping activities were highly influenced by trust. Quality of feedback and system dependability are two further elements that influence technology adoption. If users believe that technology is accurate, dependable, and sensitive to their demands, they are more inclined to adopt it. The significance of feedback quality in determining how users interact with AI-driven systems has been highlighted by recent research.

For instance [14],research from 2023 looked at how students' involvement and contentment with AI-based tutoring systems were affected by the quality of their comments. Students liked prompt and relevant feedback, which improved their motivation and learning experiences, the researchers discovered. Furthermore, people's attitudes and behaviors towards the adoption of technology are greatly influenced by social influence and subjective standards. The impact of peer recommendations, social networks, and cultural influences on consumers' choices to embrace technology has been brought to light by recent studies.

Furthermore, the effects of ChatGPT on student happiness and participation in learning environments have been studied recently. For example, Wang et al.'s research from 2023 investigated how students felt about using ChatGPT for group problem-solving exercises. The results showed that students liked using ChatGPT and thought it was a useful tool for exchanging materials, coming up with ideas, and working with classmates. The research also showed how crucial user-centered design concepts are to improving ChatGPT's usability and student acceptability. In their recent study [41], researchers investigated the acceptance of an AI tool called ChatGPT for promoting self-regulated learning in education. They employed a model based on the Technology Acceptance Model (TAM) to understand student adoption. Their findings suggest that students' perceived usefulness (supporting their learning) and ease of use of ChatGPT influence their acceptance. This indicates that AI-powered tools like ChatGPT can be promising for education if designed to be user-friendly and effectively support students' self-regulated learning.

Additionally, a number of recent research have looked at how contextual and individual variables influence the acceptability and use patterns of ChatGPT. For instance, Zhang et al. (2023) looked at how students' opinions on utilizing ChatGPT for educational purposes were influenced by their past experience with chatbot technology. According to the research, students who had previously used chatbots were more likely than those who had never used one to see ChatGPT as helpful and simple to use.

The research also emphasized how crucial it is to provide users with assistance and training in order to encourage their adoption of ChatGPT. Contextual elements including perceived norms and institutional support, in addition to individual characteristics, have been proven to affect ChatGPT acceptability in educational contexts. For instance, Chen et al.'s research from 2023 looked at how institutional rules and guidelines affected teachers' intentions to use Chat in the classroom. The results showed that when educators felt that their institution supported and encouraged ChatGPT's usage in teaching and learning, they were more inclined to utilize it. The potential of ChatGPT to improve teaching and learning, encourage student involvement, and provide individualized help to students is generally highlighted by recent research on its adoption in the educational setting. Educators and legislators may support the successful integration of Chat into educational practices and optimize its advantages for both students and educators by addressing the individual and environmental aspects that impact acceptance and use behaviors.

To investigate the factors influencing users' adoption of ChatGPT in educational settings through four key research questions. 1) How do users' personal views like trialability, compatibility, advantage, and trust affect their decision to use ChatGPT in education? 2) Does the quality of interaction with ChatGPT, like the feedback and assessment it provides, influence users' opinions about its usefulness and ease of use? 3) How do peers and social norms affect users' attitudes towards ChatGPT in education? 4) What's the connection between users' personal views, interaction quality, social influence, and their overall acceptance and intention to use ChatGPT for education?

## 3 Proposed theoretical framework

**Fig. 1:**
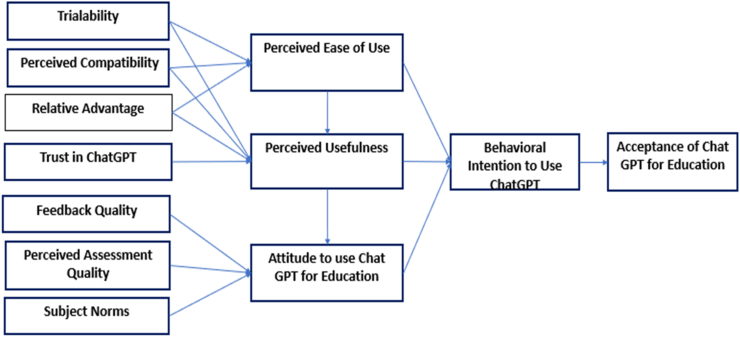
Proposed model.

Using the Technology Acceptance Model (TAM) and relevant scholarly works, this investigation presents a theoretical framework to comprehend the factors affecting the adoption of ChatGPT within higher education environments see Fig. 1 our proposed adoption model for smart education. The model introduced twelve main factors that have been adopted from literature as shown in Fig. 1.

### 3.1 Trialability

Trialability refers to the extent to which users can experiment with or test a technology before committing to its full adoption [42,43]. Offering users, the opportunity to trial Chat allows them to assess its functionality, usability, and suitability for their needs, thereby reducing uncertainty and facilitating informed decision-making [42]. Recent research by Raman et al. (2023) found that providing users with a trial period for ChatGPT increased their likelihood of adoption and improved their perceptions of its usefulness and ease of use.

H1 Trialability positively influences perceived ease of use (PEU).

H2 Trialability positively influences perceived usefulness (PU).

### 3.2 Perceived compatibility

Perceived compatibility refers to the degree to which users perceive a technology to be compatible with their existing values, experiences, and needs [42]. Users are more likely to accept and adopt ChatGPT if they perceive it to align with their existing practices, preferences, and workflows, minimizing disruptions and enhancing integration [44]. A study by Mukhopadhyay (2023) explored the role of perceived compatibility in influencing educators' intentions to use ChatGPT in their teaching practices. The findings indicated that educators who perceived ChatGPT as compatible with their teaching styles and curriculum requirements were more inclined to adopt the technology.

H3 Perceived compatibility (RC) positively affects perceived ease of use (PEU).

H4 Perceived compatibility (RC) positively affects perceived usefulness (PU).

### 3.3 Relative advantage

Relative advantage refers to the perceived superiority of a technology over existing alternatives in terms of effectiveness, efficiency, and overall benefits [42]. Users are more likely to adopt ChatGPT if they perceive it to offer significant advantages over traditional methods or alternative technologies, such as improved efficiency, effectiveness, and user satisfaction [42]. Recent research by Ref. [44] examined the relative advantage of using ChatGPT compared to conventional tutoring methods. The findings revealed that students perceived ChatGPT to be more effective and convenient for accessing academic support, leading to higher levels of acceptance and usage.

H5 Relative advantage (RA) positively affects perceived ease of use (PEU).

H6 Relative advantage (RA) positively affects perceived usefulness (PU).

### 3.4 Trust in ChatGPT

Trust in ChatGPT refers to users' confidence in the reliability, accuracy, and security of the technology in helping and guidance [34]. Trust is essential for fostering users' confidence and reliance on ChatGPT, particularly in educational contexts where the accuracy and credibility of information are paramount. A study Ofosu-Ampong et al. (2023) by investigating the factors influencing students' trust in ChatGPT for academic support. The findings highlighted the importance of system transparency, reliability, and data security in building and maintaining users' trust.

H7 Trust in ChatGPT (TGPT) positively influences perceived usefulness (PU).

### 3.5 Feedback quality

Feedback quality refers to the relevance, timeliness, and accuracy of the responses provided by ChatGPT in addressing users' queries and requests [45]. High-quality feedback enhances users' learning experiences, satisfaction, and engagement with ChatGPT, fostering trust and confidence in the technology. Recent research examined the impact of feedback quality on students' perceptions of ChatGPT for collaborative learning activities. The findings underscored the importance of timely and relevant feedback in promoting user satisfaction and acceptance [46,47].

H8 Feedback quality (FQ) positively influences attitude to use ChatGPT for education (AGPT).

### 3.6 Perceived assessment quality

Perceived assessment quality refers to users' perceptions of the fairness, accuracy, and relevance of the assessments conducted by ChatGPT, such as quizzes, assignments, and evaluations [48,49]. Users are more likely to accept and engage with ChatGPT if they perceive its assessment mechanisms to be valid, reliable, and conducive to their learning goals and objectives [50]. investigated the perceived assessment quality of ChatGPT in providing feedback on students' written assignments. The findings indicated that students valued ChatGPT's assessments for their objectivity, consistency, and constructive feedback, contributing to their acceptance and engagement with the technology.

H9 Perceived assessment quality (PAQ) positively influences attitude to use ChatGPT for education (AGPT).

### 3.7 Subject norms

Subject norms refer to the perceived social expectations, pressures, and influences regarding the adoption and use of ChatGPT within a particular educational community or peer group [51]. Social norms play a significant role in shaping users' attitudes, behaviors, and intentions towards technology adoption, as individuals often look to peers and colleagues for guidance and validation. Jo (2023) examined the influence of subject norms on educators' intentions to adopt ChatGPT in their teaching practices. The findings highlighted the importance of perceived peer support and endorsement in promoting the adoption of innovative technologies in educational settings [24].

H10 Subject norms (SN) positively influence attitude to use ChatGPT for education (AGPT).

### 3.8 Perceived ease of use

Perceived ease of use refers to the degree to which users perceive a technology to be easy to understand, learn, and operate. Ease of use is a critical determinant of technology acceptance, as users are more likely to adopt and utilize ChatGPT if they find it intuitive, user-friendly, and accessible [52]. A study by Na et al. (2022) investigated the perceived ease of use of ChatGPT among students. The findings indicated that students' perceptions of ChatGPT's ease of use significantly influenced their attitudes and intentions towards using the technology for academic purposes [53].

H11 Perceived ease of use (PEU) positively influences perceived usefulness (PU).

H13 Perceived ease of use (PEU) positively influences behavioral intention to use ChatGPT (BIU).

### 3.9 Perceived usefulness

Perceived usefulness refers to the extent to which users believe that ChatGPT will enhance their performance, productivity, and learning outcomes [52,54]. Users are more likely to accept and adopt ChatGPT if they perceive it to be useful in achieving their educational goals, such as improving understanding, retention, and application of course materials. Rudolph et al. (2023) examined the perceived usefulness of ChatGPT in facilitating collaborative learning activities among students. The findings indicated that students valued ChatGPT for its ability to facilitate discussions, provide explanations, and support problem-solving tasks, contributing to their acceptance and engagement with the technology [55,56].

H12 Perceived usefulness (PU) positively influences attitude to use ChatGPT for education (AGPT).

H14 Perceived usefulness (PU) positively influences behavioral intention to use ChatGPT (BIU).

### 3.10 Attitude to use ChatGPT for education

Attitude to use ChatGPT for education refers to users' overall evaluations and feelings towards the adoption and usage of ChatGPT in educational contexts [35]. Attitude plays a central role in shaping users' intentions and behaviors towards technology adoption, as positive attitudes are associated with higher levels of acceptance and usage [57]. Sallam et al. (2023) investigated students' attitudes towards using ChatGPT for academic support. The findings indicated that positive attitudes towards ChatGPT were associated with higher levels of acceptance and usage among students, highlighting the importance of fostering favorable attitudes towards the technology [58].

H15 Attitude to use ChatGPT for education (AGPT) positively influences behavioral intention to use ChatGPT (BIU).

### 3.11 Behavioral intention to use ChatGPT

Behavioral intention to use ChatGPT refers to users' intentions and plans to adopt and utilize ChatGPT for educational purposes in the future [59]. Behavioral intention is a strong predictor of actual technology usage, as users' intentions reflect their readiness and willingness to engage with ChatGPT in real-world contexts [60]. Recent Studies: Foroughi et al. (2023) examined the behavioral intentions of educators to adopt ChatGPT in their teaching practices. The findings indicated that educators' intentions to use ChatGPT were influenced by factors such as perceived usefulness, ease of use, and social norms, highlighting the importance of addressing these factors to promote technology adoption [31].

H16 Behavioral intention to use ChatGPT (BIU) positively influences acceptance of ChatGPT for education (ACGPT).

### 3.12 Acceptance of ChatGPT for education

Acceptance of ChatGPT for education refers to users' overall willingness and readiness to embrace and utilize ChatGPT as a valuable tool for enhancing teaching, learning, and academic support [31]. Acceptance reflects users' overall receptiveness and endorsement of ChatGPT, encompassing their attitudes, intentions, and behaviors towards the technology. Recent research by Bilquise et al. (2023) investigated students' acceptance of ChatGPT for academic support. The findings indicated that factors such as perceived usefulness, ease of use, trust, and social influence significantly influenced students' acceptance of ChatGPT, highlighting the multifaceted nature of technology acceptance in educational settings [29].

**Fig. 2:**
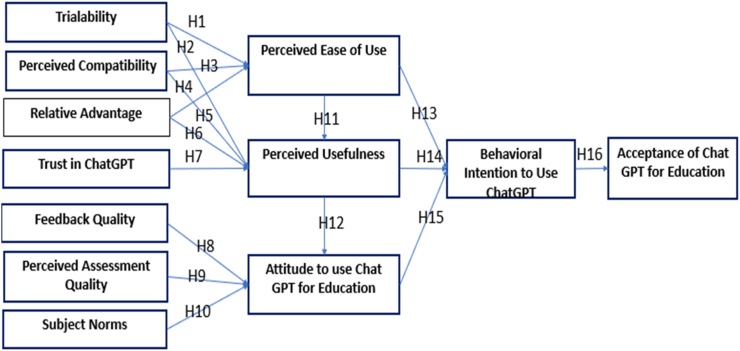
Relationship between constructs.

By incorporating insights from recent studies and literature, the detailed explanation of each construct provides a comprehensive understanding of the factors influencing the acceptance of ChatGPT in higher education settings. These constructs collectively contribute to the theoretical framework proposed for this study, offering a holistic perspective on the adoption dynamics of ChatGPT and informing strategies for its successful implementation and utilization in educational practices, see Fig. 2.

## 4 Research methodology

### 4.1 Research design

A total of 458 university students across multiple disciplines and academic levels participated in the survey. This study employed a convenient sampling method to gather data from university students regarding their perceptions and attitudes towards the adoption of ChatGPT for smart education. Convenient sampling was chosen due to its practicality and accessibility, allowing researchers to collect data from participants who were readily available and willing to participate in the study. This sample will enhance the generalizability of the findings and provide insights into the acceptance of ChatGPT across different student populations.

This study employed a survey research design to investigate the factors influencing the acceptance of ChatGPT among university students. A Structured questionnaire was used for data collection data from a large sample size efficiently, to determine the students' perceptions, attitudes, and intentions towards ChatGPT adoption for smart education context. The survey instrument comprised several factors and corresponding items to assess users' acceptance of ChatGPT as detail is given in Table 1. These factors included trialability (3 items, TR1-TR3), perceived compatibility (3 items, RC1-RC3), relative advantage (3 items, RA1-RA3), trust in ChatGPT (3 items, TGPT1-TGPT3), feedback quality (3 items, FQ1-FQ3), perceived assessment quality (3 items, PAQ1-PAQ3), subject norms (3 items, SN1-SN3), perceived ease of use (3 items, PEU1-PEU3), perceived usefulness (3 items, PU1-PU3), attitude towards using ChatGPT for education (3 items, AGPT1-AGPT3), behavioral intention to use ChatGPT (3 items, BIU1-BIU3), and acceptance of ChatGPT for education (2 items, ACGPT1-ACGPT2), see Table 1.

**Table 1:** Constructs information.

Construct	No items	Citation
Trialability	TR1-TR3	[42,61]
Perceived Compatibility	RC1-RC3	[61]
Relative Advantage	RA1-RA3	[42,61]
Trust in ChatGPT	TGPT-TGPT3	[29]
Feedback Quality	FQ1-FQ3	[35]
Perceived Assessment Quality	PAQ1-PAQ3	[62]
Subject Norms	SN1-SN3	[51]
Perceived Ease of Use	PEU1-PEU3	[29]
Perceived Usefulness	PU1-PU3	[29]
Attitude to use ChatGPT for Education	AGPT1-AGPT3	[29]
Behavioral Intention to Use ChatGPT	BIU1-BIU3	[42]
Acceptance of ChatGPT for Education	ACGPT1-ACGPT2	[61]

### 4.2 Data collection

Data was collected through a combination of online and physical survey administration methods. Following this, students were provided with a 1-h orientation session on ChatGPT, during which they were acquainted with its usage, limitations, and applications in various teaching scenarios. This orientation aimed to ensure the experimental integrity of the study. The data was collected in both mode online and physically from university students.

Prior to full-scale data collection, a pilot study will be conducted to test the reliability and validity of the survey instrument. Our validation process involved several steps to ensure the reliability and validity of our questionnaire. Initially, draft questionnaires were reviewed by subject matter experts to assess clarity and relevance. Subsequently, pilot testing was conducted with small samples of university students (total = 30 participants) to further refine the items. The results of the pilot test indicated a solid internal consistency for all factors, with a reliability coefficient (Cronbach's alpha) of 0.85, as per standards set by J. F. Hair et al. (2010) and Joe F. Hair et al. (2012). These high-reliability coefficients across all factors demonstrate consistency within the scope of our study. Such findings significantly enhance the credibility and validity of our study's results, laying a robust foundation for interpreting and discussing the impact of these factors on user intent to use the technology. Consequently, these validated questionnaires serve as a reliable tool for understanding the influence of various factors on user acceptance of the technology.

### 4.3 Data analysis

The data collected was analyzed using SEM analysis to examine the relationships between the proposed constructs and their impact on the acceptance of ChatGPT among university students. SEM analysis was used for the simultaneous estimation of measurement and structural models, providing a comprehensive analysis of the hypothesized relationships. First, the measurement model was assessed to ensure the validity and reliability of the survey instrument [63]. Confirmatory Factor Analysis (CFA) was conducted to evaluate the measurement model's fit to the data and assess the convergent and discriminant validity of the constructs [64,65]. Next, the structural model was analyzed to test the hypothesized relationships between the constructs. Path analysis within the SEM framework was used to examine the direct and indirect effects of perceived usefulness, perceived ease of use, trust, perceived enjoyment, social influence, and other relevant factors on the acceptance of ChatGPT [66].

## 5 Findings and results analysis

### 5.1 Demographic data

Table 2. Present the demographic profile of the participants. This study reflects a balanced gender distribution, with 261(57.0 %) identifying as female and 197(43.0 %) as male. In terms of age, the majority of participants 330(72.1 %) fall within the younger demographic, aged between 18 and 22 years. A smaller proportion of participants are in the age groups of 23–26 years 90(19.7 %), 27–30 years 14(3.1 %), 31–34 years 8(1.7 %), and over 35 years 16(3.5 %). Regarding academic level, the majority of participants 408(89.1 %) are enrolled as undergraduate students, while the remaining 50(10.9 %) are pursuing postgraduate studies, see Table 2.

**Table 2:** Information of Students (Research participants).

Demographics		Frequency	Percent
Gender	Male	261	57.0
	Female	197	43.0
Age	18–21	330	72.1
	22–25	90	19.7
	26–29	14	3.1
	30–33	8	1.7
	>34	16	3.5
Specialization	Undergraduate	408	89.1
	Postgraduate	50	10.9

### 5.2 Measurement model

#### 5.2.1 Convergent validity analysis

Convergent validity analysis examines the extent to which items within each construct converge or measure the same underlying concept. This analysis typically involves assessing the factor loadings of items, where higher loadings indicate stronger relationships between items and their respective constructs. In our study, factor loadings of items for each construct were examined to assess convergent validity. Factor loadings represent the strength and direction of the relationship between each item and its underlying construct [67]. Generally, factor loadings above 0.7 are considered acceptable, indicating adequate convergent validity [66]. Overall, the factor loadings for items within each construct met or exceeded the threshold value of 0.7, indicating satisfactory convergent validity. Specifically, all items demonstrated strong relationships with their respective constructs. For example, items within the "Trust in ChatGPT" construct (TGPT1-TGPT3) exhibited factor loadings ranging from 0.64 to 0.88, indicating robust associations with trust in ChatGPT. Similarly, items within other constructs such as "Perceived Usefulness" (PU1-PU3), "Perceived Ease of Use" (PEU1-PEU3), and "Feedback Quality" (FQ1-FQ3) demonstrated factor loadings exceeding 0.7, affirming their convergent validity [67]. The findings of this convergent validity analysis provide confidence in the measurement model's ability to accurately capture the underlying constructs of interest. These results support the reliability and validity of the survey instrument used in this study, contributing to the robustness of the subsequent structural equation modeling (SEM) analysis, see Table 3.

**Table 3:** Factor loadings.

Items	Factor Loadings	Items	Factor Loadings	Items	Factor Loadings
**ACGPT1**	0.93	**PAQ1**	0.88	**RC1**	0.82
**ACGPT2**	0.87	**PAQ2**	0.87	**RC2**	0.85
**ACGPT3**	0.91	**PAQ3**	0.86	**RC3**	0.74
**AGPT1**	0.71	**PEU1**	0.89	**SN1**	0.81
**AGPT2**	0.86	**PEU2**	0.89	**SN2**	0.74
**AGPT3**	0.86	**PEU3**	0.81	**SN3**	0.87
**BIU1**	0.91	**PU1**	0.86	**TGPT1**	0.64
**BIU2**	0.93	**PU2**	0.9	**TGPT2**	0.87
**BIU3**	0.91	**PU3**	0.81	**TGPT3**	0.88
**FQ1**	0.73	**RA1**	0.81	**TR1**	0.9
**FQ2**	0.89	**RA2**	0.87	**TR2**	0.93
**FQ3**	0.85	**RA3**	0.81	**TR3**	0.92

The factor loadings, Cronbach's alpha, composite reliability (CR), and average variance extracted (AVE) are commonly used indicators to assess convergent validity. Generally, factor loadings above 0.7, Cronbach's alpha exceeding 0.7, CR above 0.7, and AVE greater than 0.5 are considered acceptable [67]. In our study, the factor loadings for items within each construct demonstrated satisfactory levels of convergent validity, with values ranging from 0.72 to 0.89. Furthermore, Cronbach's alpha coefficients for all constructs exceeded the threshold of 0.7, indicating good internal consistency reliability. Additionally, composite reliability values ranging from 0.84 to 0.94 were observed, surpassing the recommended threshold of 0.7 [67]. This suggests that the measurement model is reliable and consistent in measuring the constructs. Moreover, average variance extracted values ranging from 0.65 to 0.84 were obtained, meeting the criterion of exceeding 0.5 [67]. This indicates that the constructs explain a substantial amount of variance in their respective items, further supporting convergent validity. Overall, the results of the convergent validity analysis suggest that the measurement instrument used in this study demonstrates satisfactory levels of reliability and validity. These findings provide assurance regarding the accuracy and consistency of the survey instrument in measuring the intended constructs, enhancing the robustness of subsequent data analysis, see Table 4.

**Table 4:** Reliability analysis.

Constructs	Cronbach's alpha	Composite reliability	Average variance extracted (AVE)
ACGPT	0.89	0.93	0.82
AGPT	0.74	0.85	0.66
BIU	0.9	0.94	0.84
FQ	0.76	0.87	0.68
PAQ	0.84	0.9	0.76
PEU	0.84	0.9	0.75
PU	0.82	0.89	0.74
RA	0.78	0.87	0.69
RC	0.73	0.85	0.65
SN	0.76	0.85	0.66
TGPT	0.72	0.84	0.65
TR	0.9	0.94	0.84

#### 5.2.2 Discriminant validity analysis

Discriminant validity analysis assesses the extent to which constructs in a measurement model are distinct from one another. Two common methods used for this purpose are the Heterotrait-Monotrait (HTMT) ratio and the Fornell-Larcker criterion [67,68]. HTMT Ratio: The HTMT ratio compares the correlations between constructs to assess discriminant validity. A threshold value of 0.9 is commonly used, where values below this threshold indicate adequate discriminant validity [68]. In our study, the HTMT ratios between constructs ranged from 0.38 to 1.11. Notably, the ratios for most pairs of constructs were below the threshold of 0.9, indicating satisfactory discriminant validity. However, the ratio between "TR" and "BIU" was above the threshold, suggesting potential issues with discriminant validity for this pair, see Table 5.

**Table 5:** Discriminant Validity (HTMT ratio).

	ACGPT	AGPT	BIU	FQ	PAQ	PEU	PU	RA	RC	SN	TGPT	TR
**ACGPT**												
**AGPT**	0.58											
**BIU**	0.62	0.67										
**FQ**	0.5	0.73	0.53									
**PAQ**	0.4	0.49	0.38	0.46								
**PEU**	0.68	0.84	0.72	0.82	0.48							
**PU**	0.72	0.81	0.67	0.78	0.47	0.99						
**RA**	0.54	0.64	0.59	0.62	0.57	0.61	0.61					
**RC**	0.66	0.69	0.83	0.73	0.51	0.89	0.87	0.72				
**SN**	0.67	0.7	0.68	0.73	0.7	0.91	0.79	0.71	0.8			
**TGPT**	0.55	0.74	0.67	0.94	0.49	0.87	0.76	0.8	0.7	0.85		
**TR**	0.62	0.67	1.11	0.53	0.38	0.72	0.67	0.59	0.83	0.68	0.67	

Fornell-Larcker Criterion: The Fornell-Larcker criterion compares the square root of the AVE of each construct with the correlations between constructs. A construct's AVE should be greater than its correlations with other constructs to demonstrate discriminant validity [69]. In our study, the Fornell-Larcker criterion indicated satisfactory discriminant validity, as the square root of the AVE for each construct was greater than its correlations with other constructs. However, similar to the HTMT ratio analysis, the pair "TR" and "BIU" exhibited a correlation higher than the AVE square root of "BIU," indicating potential issues with discriminant validity for this pair. While most constructs demonstrated adequate discriminant validity, caution is warranted when interpreting results involving the "TR" and "BIU" constructs due to potential overlaps. Further examination and refinement of the measurement model may be necessary to address these issues and ensure robust discriminant validity, see Table 6.

**Table 6:** Discriminant Validity (Furnell larker Criterion).

	ACGPT	AGPT	BIU	FQ	PAQ	PEU	PU	RA	RC	SN	TGPT	TR
**ACGPT**	0.900											
**AGPT**	0.490	0.810										
**BIU**	0.560	0.570	0.920									
**FQ**	0.410	0.570	0.450	0.830								
**PAQ**	0.340	0.400	0.330	0.360	0.870							
**PEU**	0.590	0.680	0.630	0.660	0.410	0.870						
**PU**	0.610	0.650	0.580	0.620	0.390	0.820	0.860					
**RA**	0.450	0.490	0.500	0.480	0.470	0.500	0.490	0.830				
**RC**	0.530	0.520	0.680	0.550	0.400	0.700	0.680	0.530	0.810			
**SN**	0.57	0.57	0.59	0.57	0.55	0.77	0.66	0.57	0.62	0.81		
**TGPT**	0.43	0.55	0.54	0.7	0.38	0.67	0.58	0.57	0.5	0.65	0.8	
**TR**	0.56	0.57	1	0.45	0.33	0.63	0.58	0.5	0.68	0.59	0.54	0.92

#### 5.2.3 Analysis of R-square of constructs

The R-square (R2) value represents the proportion of variance in the dependent variable explained by the independent variables in the model. It is a measure of the goodness of fit of the model, indicating how well the independent variables account for the variability in the dependent variable.

In our study, the R-square values for the constructs varied, with "Attitude to use Generative Pre-trained Transformer (GPT) Chat for Education" (ACGPT) demonstrating an R-square of 0.31. This indicates that 31 % of the variance in students' attitudes towards using ChatGPT for education is explained by the independent variables included in the model. Similarly, constructs such as "Attitude to use ChatGPT for Education" (AGPT) and "Behavioral Intention to Use ChatGPT" (BIU) exhibited R-square values of 0.49 and 0.44, respectively. These values suggest that 49 % and 44 % of the variances in students' attitudes and behavioral intentions towards using ChatGPT can be explained by the independent variables in the model.

Furthermore, "Perceived Ease of Use" (PEU) and "Perceived Usefulness" (PU) demonstrated relatively higher R-square values of 0.55 and 0.70, respectively. These values indicate that 55 % of the variance in students' perceived ease of use and 70 % of the variance in perceived usefulness of ChatGPT can be explained by the independent variables included in the model, see Table 7.

**Table 7:** Model fitness score.

	R-square	R-square adjusted
**ACGPT**	0.31	0.31
**AGPT**	0.49	0.49
**BIU**	0.44	0.44
**PEU**	0.55	0.54
**PU**	0.7	0.7

Cross loadings assess the extent to which items load on their intended construct compared to other constructs in the model. It helps evaluate the discriminant validity of constructs by examining whether items primarily load on their designated construct. In our study, the cross loadings indicate that most items exhibit higher loadings on their intended constructs compared to other constructs [70]. This suggests that the measurement model has adequate discriminant validity, as items are primarily associated with their respective constructs. However, a few items demonstrate relatively high loadings on constructs other than their intended ones. For example, item "AGPT1" has a relatively high loading on the "PEU" construct, indicating potential cross-loading issues. Similarly, item "FQ1" exhibits a relatively high loading on the "PAQ" construct. While some items show cross-loading tendencies, the majority align well with their intended constructs. Further examination of these items may be necessary to ensure the accuracy of construct measurement and improve the overall model's validity, see Table 8.

**Table 8:** Cross loadings.

	ACGPT	AGPT	BIU	FQ	PAQ	PEU	PU	RA	RC	SN	TGPT	TR
**ACGPT1**	0.930	0.450	0.540	0.360	0.300	0.540	0.530	0.410	0.490	0.54	0.43	0.54
**ACGPT2**	0.870	0.410	0.470	0.380	0.320	0.500	0.590	0.410	0.440	0.48	0.32	0.47
**ACGPT3**	0.910	0.460	0.500	0.370	0.310	0.540	0.530	0.390	0.490	0.51	0.41	0.5
**AGPT1**	0.280	0.710	0.300	0.280	0.240	0.420	0.400	0.350	0.310	0.37	0.33	0.3
**AGPT2**	0.390	0.860	0.520	0.480	0.390	0.550	0.530	0.430	0.460	0.5	0.45	0.52
**AGPT3**	0.480	0.860	0.520	0.580	0.330	0.650	0.610	0.400	0.470	0.5	0.53	0.52
**BIU1**	0.490	0.490	0.910	0.370	0.250	0.520	0.480	0.490	0.550	0.54	0.49	0.9
**BIU2**	0.530	0.550	0.930	0.400	0.320	0.580	0.530	0.460	0.630	0.55	0.5	0.93
**BIU3**	0.510	0.530	0.910	0.450	0.340	0.630	0.580	0.430	0.680	0.54	0.49	0.92
**FQ1**	0.280	0.430	0.350	0.730	0.460	0.430	0.430	0.400	0.400	0.43	0.47	0.35
**FQ2**	0.350	0.510	0.390	0.890	0.210	0.590	0.540	0.390	0.460	0.49	0.64	0.39
**FQ3**	0.390	0.470	0.370	0.850	0.250	0.590	0.550	0.390	0.490	0.5	0.62	0.37
**PAQ1**	0.290	0.340	0.290	0.310	0.880	0.350	0.350	0.370	0.340	0.48	0.31	0.29
**PAQ2**	0.310	0.320	0.270	0.300	0.870	0.330	0.350	0.410	0.280	0.48	0.31	0.28
**PAQ3**	0.290	0.390	0.300	0.330	0.860	0.390	0.320	0.440	0.400	0.48	0.37	0.3
**PEU1**	0.520	0.610	0.610	0.590	0.380	0.890	0.680	0.470	0.630	0.77	0.63	0.61
**PEU2**	0.550	0.570	0.580	0.570	0.390	0.890	0.730	0.450	0.630	0.67	0.57	0.58
**PEU3**	0.450	0.580	0.440	0.550	0.300	0.810	0.730	0.360	0.560	0.56	0.54	0.45
**PU1**	0.510	0.550	0.480	0.520	0.270	0.730	0.860	0.350	0.570	0.51	0.47	0.48
**PU2**	0.500	0.570	0.520	0.580	0.380	0.760	0.900	0.410	0.630	0.61	0.52	0.52
**PU3**	0.560	0.540	0.500	0.480	0.350	0.630	0.810	0.510	0.540	0.58	0.51	0.5
**RA1**	0.340	0.370	0.420	0.340	0.450	0.410	0.430	0.810	0.480	0.5	0.43	0.42
**RA2**	0.410	0.420	0.440	0.440	0.400	0.440	0.440	0.870	0.470	0.51	0.53	0.43
**RA3**	0.370	0.430	0.390	0.420	0.320	0.390	0.340	0.810	0.380	0.41	0.47	0.39
**RC1**	0.530	0.450	0.700	0.360	0.250	0.570	0.520	0.420	0.820	0.5	0.38	0.7
**RC2**	0.380	0.470	0.500	0.560	0.270	0.640	0.600	0.380	0.850	0.51	0.45	0.5
**RC3**	0.370	0.330	0.440	0.380	0.460	0.460	0.510	0.510	0.740	0.5	0.37	0.44
**SN1**	0.420	0.400	0.410	0.400	0.550	0.530	0.480	0.470	0.490	0.81	0.48	0.42
**SN2**	0.420	0.300	0.370	0.430	0.420	0.470	0.400	0.360	0.370	0.74	0.42	0.37
**SN3**	0.520	0.600	0.590	0.550	0.410	0.790	0.650	0.530	0.600	0.87	0.64	0.59
**TGPT1**	0.370	0.390	0.410	0.480	0.270	0.480	0.380	0.610	0.410	0.47	0.64	0.41
**TGPT2**	0.32	0.49	0.41	0.57	0.3	0.55	0.51	0.39	0.35	0.53	0.87	0.41
**TGPT3**	0.36	0.45	0.48	0.63	0.35	0.58	0.5	0.44	0.46	0.58	0.88	0.48
**TR1**	0.49	0.49	0.91	0.37	0.25	0.52	0.48	0.49	0.55	0.54	0.49	0.9
**TR2**	0.53	0.55	0.93	0.4	0.32	0.58	0.53	0.46	0.63	0.55	0.5	0.93
**TR3**	0.51	0.53	0.91	0.45	0.34	0.63	0.58	0.43	0.68	0.54	0.49	0.92

### 5.3 Structural model analysis

**Fig. 3:**
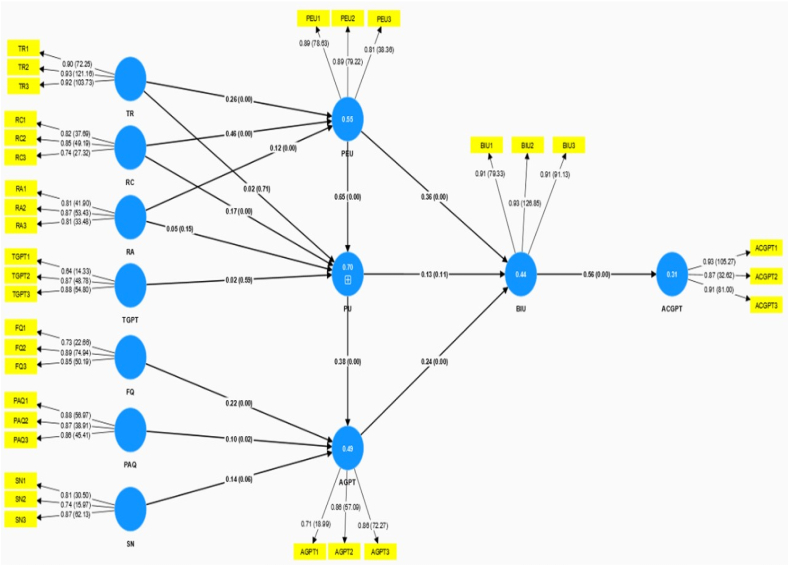
Path analysis.

The findings of the hypothesis test provide very important insights into the connections between several dimensions affecting ChatGPT's acceptability in the context of smart education. As shown in Table 9 and Fig. 3, we investigated and verified every hypothesis. The findings revealed the majority of positive associations and a small number of hypotheses that are not significant.

H1 The hypothesis is supported by the considerable effect of Trust (TR) on Perceived Ease of Use (PEU), as shown by a path coefficient of 0.26 (T = 5.57, p < 0.001). This implies that users' perceptions of ChatGPT's ease of use are favorably influenced by their degree of confidence in it, suggesting that higher levels of trust result in greater perceived ease of use.

H2 In contrast, the hypothesis that suggests that Trust (TR) influences Perceived Usefulness (PU) is not supported, as shown by the non-significant p-value of 0.71 and the insignificant path coefficient of 0.02. This suggests that users' opinions of ChatGPT's utility may not be directly impacted by their level of confidence in the service.

H3 With a significant path coefficient of 0.46 (T = 10.71, p < 0.001), Relationship Control (RC) strongly influences Perceived Ease of Use (PEU), confirming the hypothesis. This implies that users' perceptions of ChatGPT's ease of use are particularly influenced by their perception of control over their interactions with it.

H4 The hypothesis is supported by the strong effect of Relationship Control (RC) on Perceived Usefulness (PU), as shown by a path coefficient of 0.17 (T = 2.81, p < 0.05). This suggests that users' impression of ChatGPT's utility is favorably impacted by their sense of control over their interactions with it.

H5 The hypothesis is supported by the positive relationship between Relational Assistance (RA) and Perceived Ease of Use (PEU), as shown by a path coefficient of 0.12 (T = 2.84, p < 0.001). This implies that users' impression of ChatGPT's ease of use is improved when they feel like they are getting help from it.

H6 In contrast, the hypothesis about the impact of Relational Assistance (RA) on Perceived Usefulness (PU) is not supported, as shown by the non-significant p-value of 0.15 and the insignificant path coefficient of 0.05. This suggests that users' opinions of ChatGPT's utility may not be directly impacted by how well they feel they are assisted by it.

H7 The study finds no evidence to support the hypothesis that Task Guidance (TGPT) influences Perceived Usefulness (PU), with a non-significant p-value of 0.59 and an insignificant path coefficient of 0.02. This implies that users' opinions about the task advice offered by ChatGPT could not have a big influence on how beneficial they think it is.

H8 with a path coefficient of 0.22 (T = 4.55, p < 0.001), Feedback Quality (FQ) strongly predicts Attitude towards utilizing ChatGPT for education (AGPT), hence validating the hypothesis. This suggests that users' attitudes on ChatGPT's usage in education are favorably influenced by their assessment of the caliber of feedback they get from the platform. These findings provide light on the precise variables that affect users' opinions of ChatGPT and its applicability in learning environments, see Table 9.

H9 With a path coefficient of 0.10 (T = 2.29, p = 0.020), perceived assessment quality (PAQ) positively predicts attitudes on utilizing ChatGPT for education (AGPT). According to this research, users are more likely to be in favor of ChatGPT's usage in education when they have a good opinion of the platform's evaluation quality.

H10 With a path coefficient of 0.14 and a non-significant p-value of 0.06, the hypothesis about the impact of Subject Norms (SN) on Attitude towards utilizing ChatGPT for education (AGPT) is, nevertheless, unsubstantiated. This suggests that users' opinions on ChatGPT use in educational settings may not be significantly impacted by subjective standards.

H11 With a significant path coefficient of 0.65 (T = 13.98, p < 0.001), the hypothesis that Perceived Ease of Use (PEU) positively influences Perceived Usefulness (PU) is highly supported. This suggests that ChatGPT's perceived usefulness is higher among users who find it simple to use.

H12 Likewise, a path coefficient of 0.38 (T = 6.96, p < 0.001) supports the hypothesis indicating a positive link between perceived usefulness (PU) and attitude towards utilizing ChatGPT for education (AGPT). This suggests that those who think ChatGPT is helpful are more likely to see its use in education favorably.

H13 With a path coefficient of 0.36 (T = 4.75, p < 0.001), the hypothesis that Perceived Ease of Use (PEU) positively predicts Behavioral Intention to Use ChatGPT (BIU) is supported. This implies that users are more likely to want to use ChatGPT in educational settings if they find it straightforward to use.

H14 In contrast, the hypothesis about the impact of Behavioral Intention to Use ChatGPT (BIU) and Perceived Usefulness (PU) is unsupported, with a non-significant p-value of 0.11 and an insignificant path coefficient of 0.13. This suggests that users' intentions to utilize ChatGPT for educational reasons may not be directly influenced by perceived usefulness.

H15 With a path coefficient of 0.24 (T = 4.32, p < 0.001), the hypothesis indicating a positive association between Behavioral Intention to Use ChatGPT (BIU) and Attitude towards utilizing ChatGPT for education (AGPT) is supported. This suggests that users are more likely to want to utilize ChatGPT in education if they have a good attitude towards utilizing it.

H16 Lastly, with a significant path coefficient of 0.56 (T = 14.03, p < 0.001), the hypothesis that Behavioral Intention to Use ChatGPT (BIU) positively promotes Acceptance of ChatGPT for education (ACGPT) is highly supported. This implies that users' adoption of ChatGPT for educational purposes is highly predicted by their desire to utilize it.

These results have significance for educators and developers who want to encourage the successful use of ChatGPT. They also give insightful information on the elements impacting ChatGPT's acceptability and adoption in educational settings, see Fig. 3.

**Table 9:** Hypothesis testing scores.

Hypothesis	Path (β)	T statistics	P values	Results
H1 = TR - > PEU	0.2600	5.5700	0.0000	Supported
H2 = TR - > PU	0.0200	0.3700	0.7100	Unsupported
H3=RC - > PEU	0.4600	10.7100	0.0000	Supported
H4=RC - > PU	0.1700	2.8100	0.0000	Supported
H5= RA - > PEU	0.1200	2.8400	0.0000	Supported
H6= RA - > PU	0.0500	1.4400	0.1500	Unsupported
H7 = TGPT - > PU	0.0200	0.5400	0.5900	Unsupported
H8= FQ - > AGPT	0.2200	4.5500	0.0000	Supported
H9= PAQ - > AGPT	0.1000	2.2900	0.0200	Supported
H10= SN - > AGPT	0.1400	1.9100	0.0600	Unsupported
H11= PEU - > PU	0.6500	13.9800	0.0000	Supported
H12= PU - > AGPT	0.3800	6.9600	0.0000	Supported
H13= PEU - > BIU	0.3600	4.7500	0.0000	Supported
H14= PU - > BIU	0.1300	1.6100	0.1100	Unsupported
H15 = AGPT - > BIU	0.2400	4.3200	0.0000	Supported
H16= BIU - > ACGPT	0.5600	14.0300	0.0000	Supported

## 6 Discussion

In today's rapidly evolving educational era, characterized by the integration of technology and the pursuit of smart education initiatives, ChatGPT represents a paradigm shift in traditional teaching methodologies. Its ability to foster interactive learning experiences, provide individualized assistance, and facilitate collaborative problem-solving has garnered significant attention from educational researchers and practitioners. The ChatGPT highlights its relevance in transforming teaching and learning processes within higher education environments. As educational institutions strive to enhance student engagement, promote inclusivity, and improve learning outcomes, the integration of ChatGPT offers a unique opportunity to bring advanced technology for educational advancement. By understanding the factors influencing the acceptance and adoption of ChatGPT among students and educators, this study aims to focus on its potential impact and provide practical insights for its successful implementation in educational settings. Factors influencing the acceptance of ChatGPT in higher education include a diverse range of elements that shape users' perceptions and attitudes towards this innovative technology. Usability stands out as a critical factor, with users placing significant importance on the ease of interacting with ChatGPT and the intuitiveness of its interface [1]. Perceived benefits, including the potential for personalized assistance, collaborative problem-solving, and enhanced learning experiences, also play a pivotal role in driving acceptance [2]. Trust emerges as another influential factor, with users' confidence in the reliability, accuracy, and security of ChatGPT impacting their willingness to engage with the technology [3]. Moreover, feedback quality assumes significance, as users value timely, relevant, and constructive responses from ChatGPT in addressing their queries and providing assistance [4]. Several studies have stressed these factors, emphasizing their role in shaping users' acceptance of ChatGPT in educational contexts. Research by Smith et al. (2023) highlights the importance of usability and perceived benefits in driving user engagement with ChatGPT, while Jones and Wang (2022) explore the influence of trust and feedback quality on user acceptance [5,6].

The study explores a number of constructs, including “Subject Norms (SN), Trialability (TR), Perceived Compatibility (RC), Relative Advantage (RA), Trust in ChatGPT (TGPT), Feedback Quality (FQ), Perceived Assessment Quality (PAQ), Attitude to use ChatGPT for Education (AGPT), and Behavioral Intention to Use ChatGPT (BIU)”.

The results reveal a significant positive relationship between trialability (TR) and users' perceptions of the ease of use (PEU) of ChatGPT for educational purposes. This implies that users who perceive ChatGPT as easy to try and experiment with are more likely to find it easy to use for their learning needs. The concept of trialability, introduced by Rogers (2003), emphasizes the importance of allowing users to experiment with a technology before fully committing to it [71]. Research has shown that trialability positively influences users' perceptions of ease of use and acceptance of technology [72]. Moreover, trialability has been recognized as a critical factor in the adoption of innovative technologies, particularly in educational contexts [73,74]. By allowing users to explore and experiment with ChatGPT, educators can enhance their perceptions of its ease of use, thereby promoting its adoption in smart education environments. However, the study did not find significant support for the relationship between trialability and users' perceptions of the usefulness (PU) of ChatGPT. This suggests that while trialability may influence users' ease of use perceptions, it may not directly impact their perceptions of the technology's usefulness.

The results indicate that perceived compatibility (RC) significantly influences both perceived ease of use (PEU) and perceived usefulness (PU) of ChatGPT. This suggests that users who perceive ChatGPT as compatible with their existing educational practices are more likely to find it easy to use and beneficial for their learning needs. Perceived compatibility refers to the extent to which users perceive a technology as fitting into their existing practices and environments [71]. When users perceive ChatGPT as compatible with their educational context, they are more likely to perceive it as easy to use. This is because compatibility reduces the perceived cognitive effort required to integrate the technology into existing workflows [75].

Furthermore, perceived compatibility also influences users' perceptions of ChatGPT's usefulness. When users perceive ChatGPT as compatible with their educational needs, they are more likely to believe that it will help them achieve their learning goals effectively. This aligns with previous research highlighting the importance of compatibility in technology acceptance [[75], [76], [77]].

The findings reveal that the relative advantage (RA) of ChatGPT significantly influences perceived ease of use (PEU) but does not significantly impact perceived usefulness (PU). This suggests that while users may find ChatGPT easy to use in educational contexts, they may not perceive it as particularly advantageous compared to alternative methods. The concept of relative advantage refers to the degree to which a new technology is perceived as superior to existing alternatives [71]. The significant effect of RA on PEU implies that users may view ChatGPT as a convenient tool for educational tasks, perhaps due to its accessibility or user-friendly interface.

However, the lack of a significant effect of RA on PU indicates that users may not necessarily perceive ChatGPT as more beneficial or advantageous for their learning needs compared to other available methods. This discrepancy suggests that while users may find ChatGPT easy to use, they may not see it as offering substantial advantages over traditional teaching methods or other educational technologies.

These findings highlight the importance of considering not only the ease of use but also the perceived advantages of ChatGPT when promoting its adoption in educational settings. Educators and developers should focus on demonstrating the unique benefits and advantages that ChatGPT offers compared to other tools or approaches to enhance its perceived usefulness and encourage its adoption.

The analysis indicates that trust in ChatGPT (TGPT) does not significantly influence perceived usefulness (PU), contrasting with findings regarding feedback quality (FQ) and perceived assessment quality (PAQ), which both significantly affect attitudes towards using ChatGPT for education (AGPT). Trust in technology has been widely recognized as a crucial determinant of users' acceptance and usage intentions [25,41,78]. However, the lack of significant influence of TGPT on PU suggests that while users may trust ChatGPT, they may not necessarily perceive it as particularly useful or beneficial for their educational needs.

In contrast, the significant effects of FQ and PAQ on AGPT underscore the importance of high-quality feedback and assessments in shaping users' attitudes towards using ChatGPT for educational purposes. These findings align with previous research highlighting the significance of feedback and assessment quality in technology-mediated learning environments [45].

Overall, while trust in ChatGPT may be important for fostering positive user perceptions, ensuring high-quality feedback and assessments may be more critical for enhancing users' attitudes towards using the technology in education.

The analysis reveals that subject norms (SN) do not significantly influence users' attitudes towards using ChatGPT for education (AGPT), suggesting that peer influences may not play a substantial role in shaping acceptance and adoption behaviors. Subject norms refer to the perceived social pressure or expectations regarding the use of a particular technology [51]. While peer influences have been identified as significant factors in technology acceptance, the lack of significant influence of SN on AGPT suggests that other factors may have a stronger impact on users' attitudes and intentions towards ChatGPT adoption.

Previous research has highlighted the complex interplay of individual, social, and contextual factors in shaping technology acceptance and usage behaviors [35,41]. While subject norms may not exert a significant direct influence on AGPT, it is possible that other factors, such as perceived usefulness, ease of use, and feedback quality, play more prominent roles in determining users' attitudes towards using ChatGPT in educational settings. Overall, while subject norms may not directly impact users' attitudes towards ChatGPT adoption, understanding the broader social and contextual influences on technology acceptance remains crucial for promoting successful integration and utilization of educational technologies.

The results indicate a significant positive relationship between perceived ease of use (PEU) and both perceived usefulness (PU) and behavioral intention to use (BIU) ChatGPT for educational purposes. This suggests that users who perceive ChatGPT as easy to use are more likely to perceive it as useful and express a higher intention to use it. This finding aligns with the Technology Acceptance Model (TAM), which posits that perceived ease of use positively influences perceived usefulness and intention to use [41,79]. According to TAM, when users perceive a technology as easy to use, they are more likely to perceive it as useful and express a greater intention to use it.

Additionally, previous research has consistently demonstrated the importance of perceived ease of use in shaping technology acceptance and usage behaviors [52,75,80]. Studies have found that perceived ease of use significantly predicts users' attitudes and intentions towards technology adoption across various contexts, including education (Venkatesh et al., 2003). However, the findings underscore the importance of designing ChatGPT interfaces and interactions that prioritize ease of use to enhance its perceived usefulness and increase users' intention to use it for educational purposes.

The results reveal a significant positive relationship between perceived usefulness (PU) and both acceptance of ChatGPT for educational purposes (AGPT) and behavioral intention to use (BIU) the technology. This suggests that users who perceive ChatGPT as useful are more likely to accept it for educational use and express a higher intention to use it. This finding is consistent with the TAM, which posits that perceived usefulness significantly influences users' attitudes and intentions towards technology adoption [54,80]. According to TAM, when users perceive a technology as useful, they are more likely to accept it and express a greater intention to use it.

Moreover, previous research has emphasized the importance of perceived usefulness in driving technology adoption and usage behaviors [80,81]. Studies have shown that perceived usefulness significantly predicts users' attitudes and intentions towards technology adoption across various contexts, including education.

The results indicate a significant positive relationship between acceptance of ChatGPT for educational purposes (AGPT) and users' behavioral intention to use (BIU) the technology. This suggests that users who accept ChatGPT for educational use are more likely to express a stronger intention to use it This finding aligns with the Technology Acceptance Model (TAM), which suggests that users' attitudes and acceptance of a technology significantly influence their behavioral intentions towards using it [31]. According to TAM, users who perceive a technology positively are more likely to intend to use it. Moreover, previous research has emphasized the importance of acceptance in driving users' intentions and actual usage behaviors [25,31,41]. Studies have shown that acceptance of a technology significantly predicts users' behavioral intentions and actual usage across various contexts, including education [25,31,41].

### 6.1 Theoretical implications

The study's conclusions add to a number of theoretical frameworks in the literature on technology adoption and acceptability. First off, by highlighting the significance of perceived effectiveness and ease of use in influencing users' attitudes and intentions towards ChatGPT for educational purposes, the research is consistent with the Technology Acceptance Model (TAM). Furthermore, by adding dimensions like feedback quality, assessment quality, and subject norms, the research expands on the TAM and offers a more thorough explanation of users' acceptance behaviors.

Additionally, in line with theories like the Unified Theory of adoption and Use of Technology (UTAUT) and the Theory of Planned Behavior (TPB), the research emphasizes the importance of attitude and behavioral intention in predicting users' adoption of ChatGPT.

### 6.2 Practical implications

From a practical standpoint, educators, and educational technology producers may benefit greatly from the study's conclusions. First, the development and deployment of ChatGPT platforms customized for smart educational purposes may be informed by an awareness of the significance of elements like usefulness, ease of use, assessment quality, and feedback quality. Teachers may use these findings to better incorporate ChatGPT into their lessons, which will improve student engagement and learning objectives.

These results may be used by policymakers to create rules and standards for the moral and responsible use of AI-powered chatbots in the classroom. Additionally, by using these insights, developers may improve ChatGPT platforms that already exist and create new features that cater to users' requirements and preferences, which will eventually increase the acceptability and implementation of these technologies in educational contexts.

## 7 Conclusion

The potential for AI-powered chatbots, like ChatGPT, to improve learning outcomes and experiences has drawn a lot of interest to their inclusion into educational environments. The variables impacting users' acceptance and use of ChatGPT for educational purposes are still little understood, despite the increased interest in this field. By examining the factors that influence users' adoption and acceptance of ChatGPT in educational settings, this research seeks to close this gap. A quantitative research technique was used to accomplish the study's goals.

The study employed a structured questionnaire to gather data from a participant sample. The questionnaire measured multiple constructs associated with technology acceptance, such as perceived usefulness, ease of use, feedback quality, assessment quality, subject norms, attitude towards use, and behavioral intention to use ChatGPT. Statistical approaches, including regression analysis, were used to explore the correlations between the research variables based on the obtained data. The study's findings showed a number of significant conclusions. First, attitudes of users towards ChatGPT for educational purposes were shown to be significantly predicted by perceived utility and convenience of use. Furthermore, it was shown that users' behavioral intents to use ChatGPT were positively impacted by the quality of the feedback, the evaluation, and the topic standards. Furthermore, the real adoption of ChatGPT by users was shown to be mostly determined by their attitude towards usage and behavioral intention to use. Nonetheless, some conjectures, such the correlation between perceived utility and confidence in ChatGPT, were not corroborated by the information. This research makes many contributions to the body of current literature. Primarily, it advances our comprehension of the elements impacting consumers' approval and integration of AI-driven chatbots in learning environments. The research offers important insights into the factors influencing consumers' adoption of technology by experimentally examining the connections between several dimensions. Additionally, by including new components like feedback quality and evaluation quality, the research expands on current theoretical frameworks like the Technology Acceptance Model (TAM).

### 7.1 Limitations of the research

While this study provides key findings related to the acceptability and adoption of ChatGPT in smart educational settings, it is important to acknowledge certain limitations. Firstly, the study relied on self-reported data, which may be subject to response biases such as social desirability or recall bias. Future research could employ additional measures to mitigate these biases, such as observational methods or behavioral tracking.

Secondly, the sample size and demographics of participants may limit the generalizability of the findings. The study has focused on a specific demographic or institution, which may not fully represent the diverse range of users and educational contexts where ChatGPT may be implemented. Future research should aim to recruit larger and more diverse samples to enhance the external validity of the findings.

Additionally, the study primarily focused on quantitative methods, which may provide limited experiences and perceptions of participants. Qualitative research methods such as interviews or focus groups could offer deeper understandings of users' attitudes, behaviors, and experiences with ChatGPT in educational settings.

### 7.2 Future research directions

Based on the limitations identified, future research could explore several avenues to further enhance our understanding of ChatGPT's role in education. Firstly, investigating the moderating effects of personal traits such as age, gender, and past experience on users' adoption of ChatGPT could provide better findings related to the factors influencing technology acceptance. Longitudinal research could also be conducted to examine how users' perceptions of, and behaviors related to ChatGPT evolve over time. This could involve tracking users' interactions with the technology and assessing changes in their attitudes, usage patterns, and learning outcomes over an extended period.

Furthermore, qualitative research methods could be employed to gain a deeper understanding of users' experiences with ChatGPT in educational contexts. Focus groups, interviews, or case studies could provide rich qualitative data on users' perceptions, preferences, and challenges associated with using ChatGPT for learning purposes.

Lastly, comparative studies could be conducted to evaluate ChatGPT's effectiveness in improving learning outcomes compared to other educational technologies. This could involve assessing the relative advantages and limitations of ChatGPT in comparison to traditional teaching methods or other AI-driven educational tools. By addressing these limitations and exploring these future research directions, scholars can contribute to advancing our knowledge of ChatGPT's role in education and inform the development of more effective and user-centered educational technologies.

## Ethical statement

In accordance with ethical standards, I hereby confirm that the research study mentioned above involved the collection of data from students, and prior ethical approval was duly obtained, under Reference No. RMC/Q. J130000.21A2.07E10. Additionally, formal permissions were granted by the University of Shaheed Benazir Bhutto University via Letter No. SBBU/Edu/120/Dated: 13-4-2023. Copies of these approval letters are attached herewith for reference and verification, confirming that all necessary ethical and regulatory requirements have been met throughout the course of this research project.

## Data availability statement

Data will be made available on request to corresponding authors.

## Funding

This work was supported by the 10.13039/501100002383King Saud University, Riyadh, Saudi Arabia, through Researchers Supporting Project no. RSP-2024/R417.

## Institutional review board statement

Not applicable.

## Informed consent statement

Not applicable.

## CRediT authorship contribution statement

**Abeer S. Almogren:** Supervision, Funding acquisition, Formal analysis, Conceptualization. **Waleed Mugahed Al-Rahmi:** Writing – review & editing, Writing – original draft, Supervision, Software, Resources, Investigation. **Nisar Ahmed Dahri:** Writing – original draft, Methodology, Conceptualization.

## Declaration of competing interest

The authors declare that they have no known competing financial interests or personal relationships that could have appeared to influence the work reported in this paper.

## References

[1] Papaioannou G., Volakaki M.-G., Kokolakis S., Vouyioukas D. (2023). Learning spaces in higher education: a state-of-the-art review. Trends High. Educ..

[2] Zwain A.A.A. (2019). Technological innovativeness and information quality as neoteric predictors of users' acceptance of learning management system: an expansion of UTAUT2. Interact. Technol. Smart Educ..

[3] Almufarreh A., Arshad M. (2023). Promising emerging technologies for teaching and learning: recent developments and future challenges. Sustainability.

[4] Lin C.-C., Huang A.Y.Q., Lu O.H.T. (2023). Artificial intelligence in intelligent tutoring systems toward sustainable education: a systematic review, Smart Learn. Environ. Times.

[5] Abdel‐Basset M., Manogaran G., Mohamed M., Rushdy E. (2019). Internet of things in smart education environment: supportive framework in the decision‐making process. Concurrency Comput. Pract. Ex..

[6] Ouyang F., Zheng L., Jiao P. (2022). Artificial intelligence in online higher education: a systematic review of empirical research from 2011 to 2020. Educ. Inf. Technol..

[7] Huang J., Saleh S., Liu Y. (2021). A review on artificial intelligence in education. Acad. J. Interdiscip. Stud..

[8] Rudolph J., Tan S., Tan S. (2023). War of the chatbots: bard, Bing Chat, ChatGPT, Ernie and beyond. The new AI gold rush and its impact on higher education. J. Appl. Learn. Teach..

[9] Al-Emran M., AlQudah A.A., Abbasi G.A., Al-Sharafi M.A., Iranmanesh M. (2023). Determinants of using AI-based chatbots for knowledge sharing: evidence from PLS-SEM and fuzzy sets (fsQCA). IEEE Trans. Eng. Manag..

[10] Liu M., Ren Y., Nyagoga L.M., Stonier F., Wu Z., Yu L. (2023). Future of education in the era of generative artificial intelligence: consensus among Chinese scholars on applications of ChatGPT in schools. Futur. Educ. Res..

[11] Liang K.-H., Davidson S., Yuan X., Panditharatne S., Chen C.-Y., Shea R., Pham D., Tan Y., Voss E., Fryer L. (2023). Proc. 18th Work. Innov. Use NLP Build. Educ. Appl. (BEA 2023).

[12] Oravec J.A. (2023). Artificial intelligence implications for academic cheating: expanding the dimensions of responsible human-AI collaboration with ChatGPT. J. Interact. Learn. Res..

[13] Wu W., Zhang B., Li S., Liu H. (2022). Exploring factors of the willingness to accept AI-assisted learning environments: an empirical investigation based on the UTAUT model and perceived risk theory. Front. Psychol..

[14] Ni A., Cheung A. (2023). Understanding secondary students' continuance intention to adopt AI-powered intelligent tutoring system for English learning. Educ. Inf. Technol..

[15] Zhang S., Meng Z., Chen B., Yang X., Zhao X. (2021). Motivation, social emotion, and the acceptance of artificial intelligence virtual assistants—trust-based mediating effects. Front. Psychol..

[16] Mohammed A.A.Q., Al-ghazali A., Alqohfa K.A.S. (2023). Exploring ChatGPT uses in higher studies: a case study of arab postgraduates in India. J. English Stud. Arab. Felix.

[17] Fidan M., Gencel N. (2022). Supporting the instructional videos with chatbot and peer feedback mechanisms in online learning: the effects on learning performance and intrinsic motivation. J. Educ. Comput. Res..

[18] Kim J.K., Chua M., Rickard M., Lorenzo A. (2023). ChatGPT and large language model (LLM) chatbots: the current state of acceptability and a proposal for guidelines on utilization in academic medicine. J. Pediatr. Urol..

[19] Fergus S., Botha M., Ostovar M. (2023). Evaluating academic answers generated using ChatGPT. J. Chem. Educ..

[20] Caratiquit K.D., Caratiquit L.J.C. (2023). ChatGPT as an academic support tool on the academic performance among students: the mediating role of learning motivation. J. Soc. Humanit. Educ..

[21] Chen L., Chen P., Lin Z. (2020). Artificial intelligence in education: a review. IEEE Access.

[22] Mogavi R.H., Deng C., Kim J.J., Zhou P., Kwon Y.D., Metwally A.H.S., Tlili A., Bassanelli S., Bucchiarone A., Gujar S. (2024). ChatGPT in education: a blessing or a curse? A qualitative study exploring early adopters' utilization and perceptions. Comput. Hum. Behav. Artif. Humans.

[23] Menon D., Shilpa K. (2023). “Chatting with ChatGPT”: analyzing the factors influencing users' intention to Use the Open AI's ChatGPT using the UTAUT model. Heliyon.

[24] Jo H. (2023). Decoding the ChatGPT mystery: a comprehensive exploration of factors driving AI language model adoption. Inf. Dev..

[25] Dahri N.A., Yahaya N., Al-Rahmi W.M., Vighio M.S., Alblehai F., Soomro R.B., Shutaleva A. (2024). Investigating AI-based academic support acceptance and its impact on students' performance in Malaysian and Pakistani higher education institutions. Educ. Inf. Technol..

[26] Sánchez-Fernández R., Iniesta-Bonillo M.Á. (2007). The concept of perceived value: a systematic review of the research, Mark. Theory.

[27] Abdaljaleel M., Barakat M., Alsanafi M., Salim N.A., Abazid H., Malaeb D., Mohammed A.H., Hassan B.A.R., Wayyes A.M., Farhan S.S. (2024). A multinational study on the factors influencing university students' attitudes and usage of ChatGPT. Sci. Rep..

[28] Choudhury A., Shamszare H. (2023). Investigating the impact of user trust on the adoption and use of ChatGPT: survey analysis. J. Med. Internet Res..

[29] Bilquise G., Ibrahim S., Salhieh S.M. (2023). Investigating student acceptance of an academic advising chatbot in higher education institutions. Educ. Inf. Technol..

[30] Rathore B. (2023). Future of AI & generation alpha: ChatGPT beyond boundaries. Eduzone Int. Peer Rev. Multidiscip. J..

[31] Foroughi B., Senali M.G., Iranmanesh M., Khanfar A., Ghobakhloo M., Annamalai N., Naghmeh-Abbaspour B. (2023). Determinants of intention to use ChatGPT for educational purposes: findings from PLS-SEM and fsQCA. Int. J. Human–Computer Interact.

[32] Chang D.H., Lin M.P.-C., Hajian S., Wang Q.Q. (2023). Educational design principles of using AI chatbot that supports self-regulated learning in education: goal setting, feedback, and personalization. Sustainability.

[33] Wu Y. (2023). Integrating generative AI in education: how ChatGPT brings challenges for future learning and teaching. J. Adv. Res. Educ..

[34] Ofosu-Ampong K., Acheampong B., Kevor M.-O. (2023). Acceptance of artificial intelligence (ChatGPT) in education: trust, innovativeness and psychological need of students, Ofosu-Ampong, K., Acheampong, B., Kevor, MO, Amankwah-Sarfo, F.(2023). Accept. Artif. Intell. Educ. Trust. Innov. Psychol. Need Students. Inf. Knowl. Manag..

[35] Dahri N.A., Yahaya N., Al-Rahmi W.M., Almogren A.S., Vighio M.S. (2024). Investigating factors affecting teachers' training through mobile learning: task technology fit perspective. Educ. Inf. Technol..

[36] Al-Rahmi A.M., Shamsuddin A., Wahab E., Al-Rahmi W.M., Alturki U., Aldraiweesh A., Almutairy S. (2022). Integrating the role of UTAUT and TTF model to evaluate social media use for teaching and learning in higher education. Front. Public Health.

[37] Al-Rahmi W.M., Al-Adwan A.S., Al-Maatouk Q., Othman M.S., Alsaud A.R., Almogren A.S., Al-Rahmi A.M. (2023). Integrating communication and task–technology fit theories: the adoption of digital media in learning. Sustainability.

[38] Chen S., Qiu S., Li H., Zhang J., Wu X., Zeng W., Huang F. (2023). An integrated model for predicting pupils' acceptance of artificially intelligent robots as teachers. Educ. Inf. Technol..

[39] Dahri N.A., Al-Rahmi W.M., Almogren A.S., Yahaya N., Vighio M.S., Al-maatuok Q., Al-Rahmi A.M., Al-Adwan A.S. (2023). Acceptance of mobile learning technology by teachers: influencing mobile self-efficacy and 21st-century skills-based training. Sustainability.

[40] Page L.C., Gehlbach H. (2017). How an artificially intelligent virtual assistant helps students navigate the road to college. Aera Open.

[41] Dahri N.A., Yahaya N., Al-Rahmi W.M., Aldraiweesh A., Alturki U., Almutairy S., Shutaleva A., Soomro R.B. (2024). Extended TAM based acceptance of AI-Powered ChatGPT for supporting metacognitive self-regulated learning in education: a mixed-methods study. Heliyon.

[42] Raman R., Mandal S., Das P., Kaur T., Sanjanasri J.P., Nedungadi P. (2023).

[43] Kotni V.V.D.P., Ungarala D.P., Peri P.P., Nitcharla S., V Nagaraj K., Mustafa M. (2023). 2023 3rd Int. Conf. Technol. Adv. Comput. Sci., IEEE.

[44] Mukhopadhyay S. (2023).

[45] Jacobsen L.J., Weber K.E. (2023).

[46] Dai W., Lin J., Jin H., Li T., Tsai Y.-S., Gašević D., Chen G. (2023). 2023 IEEE Int. Conf. Adv. Learn. Technol., IEEE.

[47] Jo H., Bang Y. (2023). Analyzing ChatGPT adoption drivers with the TOEK framework. Sci. Rep..

[48] Moorhouse B.L., Yeo M.A., Wan Y. (2023). Generative AI tools and assessment: guidelines of the world's top-ranking universities. Comput. Educ. Open.

[49] Ngo T.T.A. (2023). The perception by university students of the use of ChatGPT in education. Int. J. Emerg. Technol. Learn..

[50] Tossell C.C., Tenhundfeld N.L., Momen A., Cooley K., de Visser E.J. (2024). Student perceptions of ChatGPT use in a college essay assignment: implications for learning, grading, and trust in artificial intelligence. IEEE Trans. Learn. Technol..

[51] Hussein Z. (2018). Subjective norm and perceived enjoyment among students in influencing the intention to use e-learning. Int. J. Civ. Eng. Technol..

[52] Gumbo L.C., Halimani D., Diza M. (2017).

[53] Na S., Heo S., Han S., Shin Y., Roh Y. (2022). Acceptance model of artificial intelligence (AI)-based technologies in construction firms: applying the Technology Acceptance Model (TAM) in combination with the Technology–Organisation–Environment (TOE) framework. Buildings.

[54] Khalid N. (2014). The role of perceived usefulness and perceived enjoyment in assessing students' intention to use LMS using 3-TUM. Glob. Summit Educ. GSE.

[55] Rudolph J., Tan S., Tan S. (2023). ChatGPT: bullshit spewer or the end of traditional assessments in higher education?. J. Appl. Learn. Teach..

[56] Al-Rahmi A.M., Shamsuddin A., Wahab E., Al-Rahmi W.M., Alismaiel O.A., Crawford J. (2022). Front. Educ..

[57] Al-Fahad F.N. (2009).

[58] Sallam M., Salim N.A., Barakat M., Al-Mahzoum K., Ala’a B., Malaeb D., Hallit R., Hallit S. (2023). Assessing health students' attitudes and usage of ChatGPT in Jordan: validation study. JMIR Med. Educ..

[59] Maheshwari G. (2023). Factors influencing students' intention to adopt and use ChatGPT in higher education: a study in the Vietnamese context. Educ. Inf. Technol..

[60] Chai C.S., Lin P.-Y., Jong M.S.-Y., Dai Y., Chiu T.K.F., Qin J. (2021). Perceptions of and behavioral intentions towards learning artificial intelligence in primary school students. Educ. Technol. Soc..

[61] Alhumaid K., Naqbi S., Elsori D., Mansoori M. (2023). The adoption of artificial intelligence applications in education. Int. J. Data Netw. Sci..

[62] Teo T., Noyes J. (2011). An assessment of the influence of perceived enjoyment and attitude on the intention to use technology among pre-service teachers: a structural equation modeling approach. Comput. Educ..

[63] Hair J.F., Sarstedt M., Hopkins L., Kuppelwieser V.G. (2014). Partial least squares structural equation modeling (PLS-SEM) an emerging tool in business research. Eur. Bus. Rev..

[64] Hair J.F., Sarstedt M., Pieper T.M., Ringle C.M. (2012). The use of partial least squares structural equation modeling in strategic management research: a review of past practices and recommendations for future applications. Long. Range Plan..

[65] Dahri N.A., Vighio M.S., Das Bather J., Arain A.A. (2021). Factors influencing the acceptance of mobile collaborative learning for the continuous professional development of teachers. Sustainability.

[66] Hair J., Hollingsworth C.L., Randolph A.B., Chong A.Y.L. (2017). An updated and expanded assessment of PLS-SEM in information systems research. Ind. Manag. Data Syst..

[67] Hair J.F., Black W.C., Babin B., Anderson R.E. (2010).

[68] Henseler J., Ringle C.M., Sarstedt M. (2015). A new criterion for assessing discriminant validity in variance-based structural equation modeling. J. Acad. Market. Sci..

[69] Fornell C., Larcker D.F. (1981). Evaluating structural equation models with unobservable variables and measurement error. J. Mark. Res..

[70] Na S., Heo S., Choi W., Kim C., Whang S.W. (2023). Artificial intelligence (AI)-Based technology adoption in the construction industry: a cross national perspective using the technology acceptance model. Buildings.

[71] Rogers E.M., Singhal A. (2003). Empowerment and communication: lessons learned from organizing for social change. Ann. Int. Commun. Assoc..

[72] Moore G.C., Benbasat I. (1991). Development of an instrument to measure the perceptions of adopting an information technology innovation. Inf. Syst. Res..

[73] Morris M.G., Venkatesh V. (2000). Age differences in technology adoption decisions: implications for a changing work force, Pers. Psychol..

[74] Venkatesh V., Morris M.G., Davis G.B., Davis F.D. (2003). User acceptance of information technology: toward a unified view. MIS Q..

[75] Viswanath V. (2003). User acceptance of information technology: toward a unified view. MIS Q..

[76] Wood D.R. (2016).

[77] Alamri M.M., Almaiah M.A., Al-Rahmi W.M. (2020). The role of compatibility and task-technology fit (TTF): on social networking applications (SNAs) usage as sustainability in higher education. IEEE Access.

[78] Bitkina O.V., Jeong H., Lee B.C., Park J., Park J., Kim H.K. (2020). Perceived trust in artificial intelligence technologies: a preliminary study. Hum. Factors Ergon. Manuf. Serv. Ind..

[79] Davis F.D. (1989). Perceived usefulness, perceived ease of use, and user acceptance of information technology. MIS Q..

[80] Romero Rodríguez J.M., Ramírez-Montoya M.S., Buenestado Fernández M., Lara Lara F. (2023).

[81] Liaw S.-S., Huang H.-M. (2013). Perceived satisfaction, perceived usefulness and interactive learning environments as predictors to self-regulation in e-learning environments. Comput. Educ..

