# Supplementary material for: Exploring factors influencing the acceptance of ChatGPT in higher education: A smart education perspective
Source: Heliyon. 2024 May 25;10(11):e31887. doi: 10.1016/j.heliyon.2024.e31887 (PMC11154614; doi:10.1016/j.heliyon.2024.e31887)
Supplement: Multimedia component 1 [file mmc1.docx]

| **Construct** | **Survey Item** |
| --- | --- |
| **Trialability** | 1. I would like to have the opportunity to try out GPT Chat before fully committing to it. |
|  | 2. Testing GPT Chat in a few classes before implementing it campus-wide would be beneficial. |
|  | 3. I believe that having a trial period for GPT Chat would help in its adoption. |
| **Perceived Compatibility** | 4. GPT Chat aligns well with the goals and objectives of my courses. |
|  | 5. Integrating GPT Chat into my teaching methods fits well with my teaching style. |
|  | 6. I think GPT Chat is compatible with the existing technologies used in my classes. |
| **Relative Advantage** | 7. Using GPT Chat would provide better learning experiences compared to traditional methods. |
|  | 8. GPT Chat offers advantages that other educational tools do not. |
|  | 9. I believe GPT Chat would improve student engagement more than other tools. |
| **Trust in GPT Chat** | 10. I trust the accuracy of responses provided by GPT Chat. |
|  | 11. I have confidence in the security measures of GPT Chat to protect my information. |
|  | 12. I trust that GPT Chat can effectively assist me in my educational tasks. |
| **Feedback Quality** | 13. The feedback provided by GPT Chat is helpful for my learning. |
|  | 14. GPT Chat gives timely and relevant feedback on my queries. |
|  | 15. I find the feedback from GPT Chat to be accurate and reliable. |
| **Perceived Assessment Quality** | 16. GPT Chat's assessment of my work is fair and unbiased. |
|  | 17. I trust the assessment criteria used by GPT Chat to evaluate my performance. |
|  | 18. GPT Chat's assessments provide me with valuable insights into my progress. |
| **Subject Norms** | 19. My peers and colleagues consider using GPT Chat for educational purposes. |
|  | 20. There is a social expectation among students and faculty to adopt GPT Chat. |
|  | 21. I perceive that others in my academic community are supportive of using GPT Chat. |
| **Perceived Ease of Use** | 22. Using GPT Chat for educational purposes is easy for me. |
|  | 23. I find it simple to navigate and interact with GPT Chat. |
|  | 24. Learning how to use GPT Chat effectively is not complicated. |
| **Perceived Usefulness** | 25. GPT Chat enhances my learning experience. |
|  | 26. I believe that GPT Chat helps me to better understand course materials. |
|  | 27. Using GPT Chat improves my productivity in educational tasks. |
| **Attitude to use GPT Chat** | 28. I have a positive attitude towards using GPT Chat in my education. |
|  | 29. I enjoy using GPT Chat for educational purposes. |
|  | 30. I perceive using GPT Chat as beneficial for my academic progress. |
| **Behavioral Intention to Use** | 31. I intend to continue using GPT Chat for educational purposes in the future. |
|  | 32. I plan to use GPT Chat regularly in my academic activities. |
|  | 33. I am likely to recommend GPT Chat to others for educational use. |
| **Acceptance of GPT Chat** | 34. Overall, I am satisfied with using GPT Chat for educational purposes. |
|  | 35. I believe GPT Chat has the potential to positively impact higher education. |
|  | 36. I see GPT Chat as a valuable addition to the educational tools available to me. |
